# *Toxoplasma gondii* Recombinant antigen AMA1: Diagnostic Utility of Protein Fragments for the Detection of IgG and IgM Antibodies

**DOI:** 10.3390/pathogens9010043

**Published:** 2020-01-05

**Authors:** Bartłomiej Ferra, Lucyna Holec-Gąsior, Justyna Gatkowska, Bożena Dziadek, Katarzyna Dzitko

**Affiliations:** 1 Department of Molecular Biotechnology and Microbiology, Faculty of Chemistry, Gdańsk University of Technology, Narutowicza 11/12, 80-233 Gdańsk, Poland; lucholec@pg.edu.pl; 2 Department of Immunoparasitology, Faculty of Biology and Environmental Protection, University of Lodz, Banacha 12/16, 90-237 Łódź, Poland; justyna.gatkowska@biol.uni.lodz.pl (J.G.); bozena.dziadek@biol.uni.lodz.pl (B.D.); katarzyna.dzitko@biol.uni.lodz.pl (K.D.)

**Keywords:** ELISA, serological detection, apical membrane antigen 1 (AMA1), IgG, IgM, *Toxoplasma gondii*

## Abstract

*Toxoplasma gondii* is an important zoonotic protozoan that infects a wide variety of vertebrates as intermediate hosts. For this reason, the diagnosis of this disease is very important and requires continuous improvement. One possibility is to use recombinant antigens in serological tests. Apical membrane antigen 1 (AMA1), a protein located in specific secretory organelles (micronemes) of *T. gondii*, is very interesting in regard to its potential diagnostic utility. In the present study, we attempted to identify a fragment of the AMA1 protein with a high sensitivity and specificity for the serological diagnosis of human toxoplasmosis. The full-length AMA1 and two different fragments (AMA1N and AMA1C) were produced using an *Escherichia coli* expression system. After purification by metal affinity chromatography, recombinant proteins were tested for their utility as antigens in enzyme-linked immunosorbent assays (ELISAs) for the detection of IgG and IgM anti-*T. gondii* antibodies in human and mouse immune sera. Our data demonstrate that the full-length AMA1 recombinant antigen (corresponding to amino acid residues 67–569 of the native protein) has a better diagnostic potential than its N- or C-terminal fragments. This recombinant protein strongly interacts with specific anti-*T. gondii* IgG (99.4%) and IgM (80.0%) antibodies, and may be used for developing new tools for diagnostics of toxoplasmosis.

## 1. Introduction

*Toxoplasma gondii* is an obligate intracellular protozoan parasite and an important zoonotic pathogen that is capable of invading and replicating within a wide variety of nucleated host cells. This parasite infects up to 80% of the population in some regions of the world. In most cases, immunocompetent individuals are protected by both cell-mediated and humoral immune mechanisms, and, for this reason, *T. gondii* infection does not cause severe illness. These individuals remain chronically infected for the rest of their lives, with the parasites encysted in their brain and muscles, and develop life-long protective immunity against reinfection. However, *T. gondii* infection can cause life-threatening disease during pregnancy and in immunocompromised individuals [1,2]. A primary *T. gondii* infection and subsequent transplacental transmission during pregnancy can result in miscarriage or congenital defects in the infant [3]. In patients with severe immune dysfunction, reactivation of the infection produces neurological manifestations or even fatal toxoplasmic encephalitis (TE) due to active proliferation of the parasite within the brain [4]. *T. gondii* infection is also an important problem in animal breeding because it can cause the death of many fetuses in cattle and sheep [5].

Since the course of a *Toxoplasma* infection is generally asymptomatic, the diagnosis is generally based on serological tests, which depend on the types of antigens and sensitivity of the detection method (e.g., chemiluminescence and absorbance). The enzyme-linked immunosorbent assay (ELISA) for the detection of specific immunoglobulins (Igs) is an easy test to perform, and many manual and automated systems are commercially available. In nearly all of these tests, various preparations of tachyzoite antigens are used, which might be contaminated by nonparasitic material and may vary in their antigenic content due to the different preparation methods utilized. For this reason, for more than three decades, recombinantly produced *T. gondii* antigens have been considered as an alternative tool to replace the tachyzoite native antigens in the serological detection of toxoplasmosis [6,7]. To date, many different genes encoding proteins of this parasite have been cloned into bacterial and eukaryotic expression systems and have been used for the detection of specific antibodies in human and animal sera with the use ELISA or Western blotting [6,7,8,9,10]. These serological tests are based on single recombinant proteins, protein mixtures and, more recently, newer tools, such as chimeric antigens. Furthermore, the diagnostic usefulness of some preparations of recombinant antigens in determining IgG avidity assays has been estimated [6,11,12,13,14,15,16,17]. Despite the very promising results described in numerous studies, new antigenic proteins of *T. gondii* with potential diagnostic utility are still being researched and tested.

*T. gondii* is a member of the phylum Apicomplexa and contains three unique sets of secretory organelles, micronemes, rhoptries, and dense granules, which play distinct roles during and after host cell invasion [18,19,20]. Antigens located in these specific secretory organelles are very interesting in regard to their potential diagnostic utility. Micronemes are located at the apical end of the parasite and contain proteins involved in host cell recognition and attachment. The invasion process by *T. gondii* involves a moving junction (MJ) formed between the apex of the parasite and the host cell membrane [21,22]. The apical membrane antigen 1 (AMA1) belongs to the microneme group of proteins, and its production depends on the intracellular concentration of calcium ions. The current understanding is that microneme proteins (MIC; >20 antigens) are released upon first contact with the host cells. These proteins are involved in host cell recognition and attachment [23]. MIC antigens form adhesive complexes or occur as single proteins. Their functions include adhesion and disturbance of the integrity of the host cell membrane, which allows penetration of the parasite into the host cell. The molecular characterization of MICs has shown that they possess adhesive motifs usually found in higher eukaryote proteins [24]. Functional roles, including host cell attachment, motility, invasion, and a synergistic role in the infectious process, have been demonstrated for these antigens [25,26,27,28,29,30,31]. Three distinct microneme protein complexes have been identified to date: MIC6-MIC1-MIC4, MIC8-MIC3, and MIC2-M2AP. The MIC6-MIC1-MIC4 and MIC8-MIC3 complexes are responsible for targeted adhesion to the surface receptors of the target cells, allowing the creation of a connection between the parasite and the host cell. Microneme proteins that build the MIC2-M2AP complex also play a fundamental role in the movement and penetration of the parasite into the host cells. As mentioned above, the majority of MIC proteins form complexes with each other or occur individually. An exception to this rule is the AMA1 antigen. The AMA1 antigen interacts with rhoptry proteins (ROPs), namely rhoptry neck proteins (RONs), and probably forms one of the most important adhesive complexes, the so-called MJ complex, built of antigens including AMA1, RON2, RON4, RON5, and RON8 [32,33]. However, studies of the parasite lacking a functional *ama1* gene have shown that it still has the ability to penetrate into the host cell. What is more, the lack of AMA1 also affects the level of RON secretion, although the mechanism of this phenomenon is not fully understood. Nevertheless, from a diagnostic point of view, the AMA1 antigen appears to be very interesting, and may be useful for detecting the early stages of *T. gondii* invasion.

In this study, for the first time, the full-length AMA1 and two different fragments (AMA1N and AMA1C) of the apical membrane antigen were evaluated as antigenic preparations for the detection of IgG and IgM anti-*T. gondii* antibodies in human and mouse immune sera. 

## 2. Results

### 2.1. Expression and Purification of the Recombinant AMA1 Antigens

The three variants of AMA1 protein (AMA1N, AMA1C, and full-length AMA1) were expressed as insoluble proteins with calculated molecular masses of 32.07, 35.91, and 61.29 kDa, respectively. The proteins were purified using one-step metal affinity chromatography with Ni^2+^ bound to iminodiacetic acid-agarose (Novagen). The expression system produced approximately 33, 31, and 15 mg of purified proteins from a liter of induced *Escherichia coli* culture for AMA1N, AMA1C, and AMA1, respectively. The purification resulted in electrophoretically homogeneous protein preparations with a purity above 95% (results not shown).

### 2.2. Immunoreactivities of the Recombinant AMA1 Antigens with Mouse Sera

**Figure 1:**
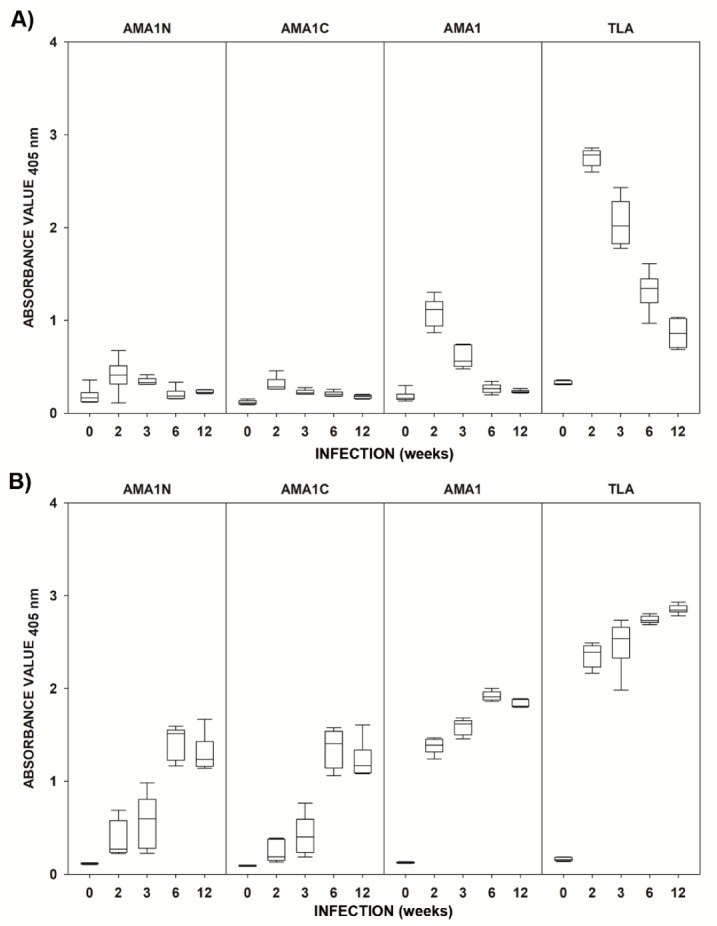
The antibody response of BALB/c mice infected with *Toxoplasma gondii* DX, tested by an IgM enzyme-linked immunosorbent assay (ELISA) (**A**) and IgG ELISA (**B**) (ꟷ min–max, □ 25%–75%, − median). Uninfected animals were marked as time “0”. The calculated cut-off values were 0.362 (IgM) and 0.130 (IgG) for AMA1N, 0.160 (IgM) and 0.107 (IgG) for AMA1C, 0.298 (IgM) and 0.141 (IgG) for apical membrane antigen 1 (AMA1), and 0.202 (IgM) and 0.370 (IgG) for the *T. gondii* native lysate antigen (TLA).

As shown in Figure 1, mouse immune sera displayed a typical reactivity with the native antigen, characterized by a drop in IgM antibody titers accompanied by the rise of IgG immunoglobulins during the course of *T. gondii* invasion. Both antibody classes were detected in mouse immune sera, using the *Toxoplasma* lysate antigen (TLA) assay, as early as 2 weeks post-invasion and their levels remained statistically higher, compared to negative controls, regardless of the phase of infection (Table 1). Out of the three tested AMA1 fragments, the full-length antigen proved most reliable in detecting specific IgM antibodies, especially during acute toxoplasmosis (2 weeks post-invasion), and had the highest absorbance values in the ELISA. The use of the other two fragments resulted in low absorbance values, although in the case of AMA1C, the reactivity of IgM antibodies in immune sera was statistically higher compared to controls at all tested time points after *T. gondii* infection. All AMA1 preparations allowed for the detection of specific serum IgG antibodies with optical density (OD) values significantly higher compared to uninfected control sera as early as 2 weeks post-invasion (Table 1). The concentrations of IgG immunoglobulins recognizing each AMA1 fragment increased during the course of infection, reaching their peak 6 weeks after *T. gondii* infection. Although the kinetics of IgG reactivity for all three antigens were similar, the highest reactivity was noted again in the case of AMA1, especially while comparing the absorbance values obtained in acute toxoplasmosis (2 and 3 weeks post invasion).

### 2.3. IgG ELISA Using Human Serum Samples

**Figure 2:**
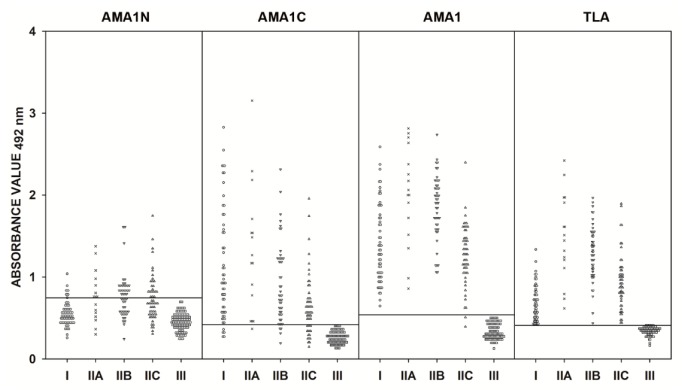
Comparison of immunoreactivity in IgG ELISA using AMA1N, AMA1C, and AMA1 proteins, as well as TLA, with sera from patients with suspected acute (I) and chronic *T. gondii* infection (IIA–IgG titer > 300 IU/mL, IIB–IgG titer between 101 and 300 IU/mL, and IIC–IgG titer ≤ 100 IU/mL), and from seronegative individuals (III). The horizontal lines represent the cut-off values.

Human serum samples from group I (sera from patients with a suspected acute phase of *T. gondii* infection), group II (sera from patients in the chronic phase of toxoplasmosis), and group III (sera from seronegative individuals) were examined by IgG ELISAs utilizing the AMA1N, AMA1C, and AMA1 recombinant proteins and TLA. The 95 sera from group III were used to determine the specificity of the IgG ELISAs. None of these sera were reactive based on the IgG ELISA cut-off value, thereby demonstrating a specificity of 100% (Figure 2). The high specificity of our IgG ELISA was confirmed by ROC analysis (Table 2). The calculated cut-off values are very similar to those obtained using the ROC analysis. However, we identified only a weak positive Pearson correlation between any of the recombinant proteins and TLA (0.104, 0.195, and 0.176 for AMA1N, AMA1C, and AMA1, respectively) (Table 3). The only statistically significant correlation was for AMA1C (*p* < 0.039).

The sensitivities of the IgG ELISAs based on the AMA1N, AMA1C, and AMA1 recombinant antigens varied for each protein preparation and were 34.6%, 84.6%, and 99.4%, respectively, for all of the positive serum samples (Figure 2, Table 2). Differences between sensitivities were observed only for the full-length AMA1 recombinant protein, with 98.9% for the standard calculation cut-off value and 99.4% using the ROC analysis. Further considerations are based on the results obtained with the ROC analysis. By analyzing the results obtained for each protein preparation, statistically significant differences (p < 0.001) were found between results from patients with suspected acute and chronic *T. gondii* infections compared to the control group (Table 4). When looking at the result obtained from group I sera (suspected acute *T. gondii* infection), we noted that the highest median value (1.324) was calculated for the IgG ELISA using the recombinant full-length AMA1 protein (Table 4). Nevertheless, this protein preparation showed a weak correlation with TLA (*r* = 0.382, *p* = 0.004). Furthermore, the individual results obtained for the AMA1 protein were significantly higher than for TLA (Table 3). Sensitivities of 100% were reported for TLA and AMA1 IgG ELISAs and a slight decrease was observed in the case of AMA1C IgG ELISA (90.9%), while the AMA1N was inadequate for the detection of specific IgG antibodies (12.7%) (Figure 2). A similar result was observed for group II serum samples (chronic *T. gondii* infection), regardless of IgG antibody titers (IIA-C). In the case of the IIA subgroup (IgG > 300 IU/mL), all protein preparations demonstrated the highest reactivity, with values of 56.3%, 93.8%, and 100% for AMA1N, AMA1C, and AMA1, respectively (Figure 2). Additionally, this is also reflected in the observed median values calculated for the IgG ELISAs based on recombinant AMA1N (0.755), AMA1C (1.226), and full-length AMA1 (2.035) proteins (Table 4). For group IIB (IgG between 101–300 IU/mL), the estimated reactivity was 51.0%, 89.8%, and 100% for AMA1N, AMA1C, and AMA1, respectively (Figure 2). The highest median value was again seen with AMA1 (1.919), but for the other two recombinant proteins, the values were almost identical, reaching 0.779 (AMA1N) and 0.774 (AMA1C) (Table 4). A decrease in reactivity was observed for the IIC group (≤100 IU/mL). In the case of the full-length AMA1 protein, this decrease was small and it was due to the fact that one serum sample with IgG antibody titers of 20 IU/mL was not correctly identified, resulting in a reactivity of 98.2%. Significant decreases in reactivities were observed for AMA1N (41.8%) and AMA1C (74.6%) (Figure 2). Regardless of the subgroup in the IgG ELISA test, the reactivity of the TLA preparation was 100%. Taking into account all statistical analyses (median, ROC analysis, and Pearson correlation), the best recombinant antigen for detecting IgG antibodies by ELISA is full-length AMA1 (AUC = 0.998, *r* = 0.793, *p* < 0.001) (Figure 2, Table 2, Table 3, and Table 4).

### 2.4. IgM ELISA Using Human Serum Samples

**Figure 3:**
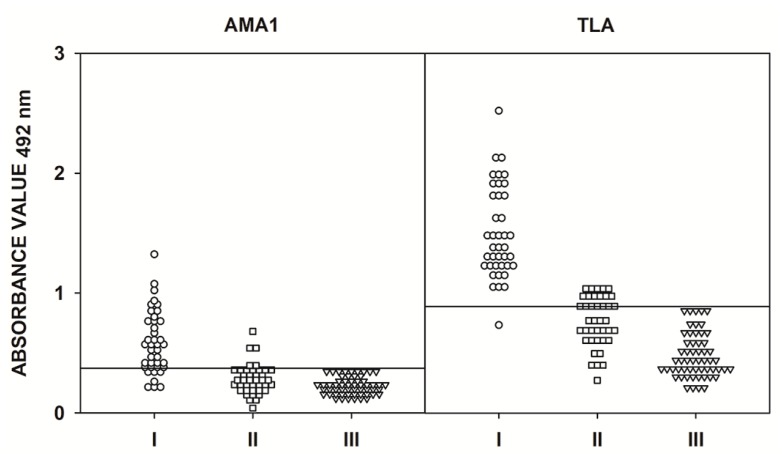
Comparison of immunoreactivity in IgM ELISA using the AMA1 protein and TLA with sera from patients with suspected acute (I – IgM +, IgG +, low avidity) and chronic *T. gondii* infection (II – IgM –, IgG +, high avidity), and from seronegative individuals (III). The horizontal lines represent the cut-off values.

A total of 156 sera from groups I, II, and III were tested. The IgM ELISA was conducted using the recombinant full-length AMA1 protein and TLA. Twenty sera from group III were tested in the IgM ELISA in order to calculate the cut-off values. The remaining 56 serum samples, none of which reacted above the cut-off values, were used to determinate the specificity of the assays. In determining assay specificity, the results obtained from group II, which included sera taken from patients with chronic *T. gondii* infection (IgM –; IgG +; high avidity), were also taken into account. Unfortunately, some of the group II sera reacted above the calculated cut-off values, which resulted in a decreased specificity of the IgM ELISA (Figure 3). However, ROC analysis showed that the cut-off value was similar for the recombinant AMA1 protein (0.372 vs. 0374), while a difference was noted for the TLA preparation (0.848 vs. 1.043). Further analysis of the results generated cut-off values using ROC analysis (0.374 and 1.043 for AMA1 and TLA, respectively). Taking all the results into account, the specificity for the TLA preparation was 100%, while for the recombinant AMA1 protein, it was estimated to be 93.8%. By analyzing the data for the AMA1 protein preparation, a statistically significant difference (*p* < 0.001) was found between the results obtained for serum samples with the presence of IgM antibodies (suspected acute phase of *T. gondii* infection) compared to the control group (Table 4). The sample distribution analysis for the recombinant AMA1 antigen and TLA generated area under the curve (AUC) values of 0.9178 and 0.9919, respectively. Moreover, the results for these recombinant proteins showed a strong positive Pearson correlation with TLA, where *r* = 0.781 (*p* < 0.001) (Table 3). However, the IgM ELISA using AMA1 displayed a reduced sensitivity, reaching 80.0%, compared to 95.0% for TLA.

## 3. Discussion

Currently, the diagnosis of *T. gondii* invasion is mainly based on the use of native antigens in enzyme-linked immunosorbent assays, which can detect IgG, IgM, and IgA antibody classes. However, in some cases, the tests yield ambiguous results. For this reason, many research groups are currently working on new diagnostic tools, mainly involving recombinant proteins. Compared to the native antigens, their production is much easier, cheaper, faster, and safer. An additional advantage of the recombinant proteins is their ability to standardize assays, as well as the possibility of selecting proteins characteristic for the developmental form of the interest of the parasite, potentially allowing for differentiation between phases of the disease. Knowledge of the complex antigenic structure of the parasite provides hope in the search for new diagnostic tools. Unfortunately, despite the fact that during the last 30 years, we have gained insight into the antigenic structure of the parasite, we still have not found the perfect antigen to detect *T. gondii* infection [6,7]. However, methods of genetic engineering and molecular biology are offering new possibilities in the development of novel antigenic recombinant proteins.

In this study, the full-length AMA1 protein and two different fragments of AMA1 were produced recombinantly, and their performance in detecting specific IgG and IgM antibodies in human and mouse sera was evaluated.

AMA1, a micronemal integral membrane protein, is the putative ligand in the MJ and build complex with RON proteins (RON2, RON4, RON5, and RON8) [32]. Therefore, undoubtedly, AMA1 plays a central role during the invasion of *T. gondii*, and thus promotes tachyzoite replication [34]. For these reasons, this protein appears to be a very interesting antigen for diagnostic and vaccine applications.

The characterization of MIC antigens indicates that they are all interesting proteins with diagnostic potential. However, until now, only a few of the more than 20 known MICs have been studied for their utility in diagnostics for toxoplasmosis. The justification for this may be the complex structure of these antigens, problems with the selection of appropriate immunodominant fragments, and difficulty in the production of functional recombinant proteins or fragments of these proteins in commonly used prokaryotic expression systems. To date, an Italian research team has obtained several MIC antigens and evaluated them for diagnostic purposes [16,35,36,37]. In one of these studies, they describe the potential diagnostic use of six different fragments of MIC proteins, including MIC2_157-235_, MIC2_466-610_, MIC3_233-307_, MIC4_53-222_, M2AP_37-263_, and AMA1_263-457_ [37]. All of these recombinant proteins were tested in an IgG ELISA using infant serum samples from mothers with primary *T. gondii* infection. In that study, the smallest diagnostic potential in newborns with *T. gondii* infection was exhibited by fragments of the MIC4 (49%) and AMA1 (57%) proteins. The reactivity of the other proteins was between 83% and 97%; however, these proteins also had a high ability to detect IgG antibodies in some percentage of uninfected newborns. This was likely the result of transfer of the maternal transition of IgG antibodies to the fetus. Selected recombinant proteins (MIC2_157-235_, MIC2_466-610_, MIC3_233-307_, and M2AP_37-263_) were also tested for their ability to detect specific IgM antibodies. The highest reactivity allowing correct diagnosis of the invasion phase was seen with the MIC2_157-235_ recombinant protein (60%). In addition, the same research team showed that a fragment of the MIC3_233-307_ antigen enabled much better determination of the avidity index [16]. Another important report describes the results obtained when the MIC1 antigen was examined. In this study, the following recombinant proteins were obtained: MIC1ex2 (amino acid residues 25–182), MIC1ex34 (amino acid residues 183–456), and MIC1 (amino acid residues 25–456) [38]. Proteins tested in the IgG ELISA demonstrated the ability to detect specific antibodies in the serum samples of patients with suspected acute toxoplasmosis (96.1% for MIC1ex2, 100% for MIC1ex34, and MIC1). However, the authors only demonstrated the usefulness of MIC1ex2 in the diagnosis of the chronic phase of toxoplasmosis (75%), while MIC1ex34 and MIC1 showed a significantly lower reactivity at the level of 52.7% and 36.1%, respectively.

In the present study, further work on the potential usefulness of the recombinant microneme antigens in the serodiagnosis of toxoplasmosis has been described. For the first time, two different fragments and the full-length AMA1 protein were tested in both IgG and IgM ELISA tests using human sera. Moreover, an assessment of humoral immune response dynamics during murine experimental toxoplasmosis was performed using recombinant *T. gondii* AMA1 antigens. Our results indicate that only the full-length protein enables a demonstration of the dynamics of the induced immune response, involving the synthesis of antigen-specific IgM antibodies, which appear and achieve maximal levels in the acute stage of *T. gondii* invasion. A substantial decrease in IgM antibodies can be observed in the second and third week after infection, when acute toxoplasmosis transitions to the chronic phase. At this point of the parasite invasion, the production of IgM and IgG class antibodies is switched, with IgG antibodies becoming the predominantly synthesized form [39,40,41]. Furthermore, the results allow us to conclude that this recombinant protein may only be useful for detecting early IgM. This hypothesis is partially confirmed by the results obtained using the IgM ELISA test with human serum samples. When testing sera samples obtained using the mouse model of toxoplasmosis, it was observed that both the C- and N-terminal end of the AMA1 antigen is characterized by the ability to interact with anti-*T. gondii* IgG antibodies from the chronic phase of toxoplasmosis. In contrast, the full-length AMA1 protein exhibits a high reactivity with specific IgG antibodies, regardless of the phase of the infection. However, in IgG ELISAs of human serum samples, it has been shown that the N-terminal end of the AMA1 protein is diagnostically inadequate, and the C-terminal end can be recognized by IgG antibodies in the vast majority of sera correctly, while the full-length AMA1 protein was characterized as having the highest reactivity. In order to understand the above results, reference should be made to the structure of the AMA1 antigen and its function. For the N-terminal fragment of the AMA1 protein, which corresponds to the first domain (DI) of the native antigen (amino acid residues 67–287), a low ability to interact with specific anti-*T. gondii* antibodies may be expected. This is due to the fact that the DI of the AMA1 antigen is mainly responsible for binding to the RON2 protein, which occurs at the beginning of the construction of the full MJ complex [33,42]. Therefore, DI likely has a limited ability to stimulate the immune system and thus to be recognized by specific antibodies. During the construction of the C-terminal end and full-length AMA1 protein, we decided not to leave the transmembrane domain (TM) and cytoplasmic tail (CT). Therefore, a C-terminal protein fragment composed of amino acid residues 287–569 corresponds to the second (DII – amino acid residues 288–415) and third (DIII – amino acid residues 416–487) domain, as well as to TM and CT. In agreement with previous studies in which a fragment of the AMA1 antigen corresponding to amino acid residues from 263–457 was used [37], our assumption was proven to be correct and it allowed us to increase the reactivity of this fragment of the protein. However, in earlier work, the protein corresponding to the entire DIII was not used for research. Therefore, it should be considered that the fragment of the AMA1 antigen containing the amino acid residues 458–487 may contain important B-cell epitopes recognized by the majority of individuals. Such a hypothesis may be relevant, especially if both DII and DIII do not interact with other proteins and constitute a connection between the parasite cells and the rest of the MJ complex. In light of our current understanding, this hypothesis also seems to confirm the fact that during penetration into the host cell, the MJ complex is cut off at amino acid residues 487–488 by rhomboid protease ROM4 [34,43,44]. It is assumed that the severed part of the MJ complex remains on the surface of the host cell and allows the penetration of other parasite cells (this mechanism is not fully understood). It is also not known whether the whole complex or only a part of it remains on the surface of the host cell [45]. Nevertheless, this suggests that after invasion, DIII of the AMA1 antigen is the most exposed and, thus, may stimulate the immune system, resulting in the synthesis of specific antibodies. On the other hand, perhaps the AMA1 antigen model developed on the basis of crystallographic and modeling studies is not entirely correct and the structure of the AMA1 protein looks different. Another possibility is that the sequence of the TM and CT also contains important epitopes recognized by specific antibodies. The possibility of another construction of the MJ complex is discussed in several works, in which it is suggested that the AMA1 antigen is responsible for direct adhesion to the host cell, and another hypothesis assumes that the binding between AMA1 and RON2 looks completely different [46,47]. The results presented so far do not exclude any of these possibilities, confirming that further research on the AMA1 antigen, as well as the MJ complex, is necessary. Similar to other *T. gondii* recombinant proteins [6,7], the results demonstrated that the full-length AMA1 protein is characterized by the highest reactivity in immune mouse and human sera. Taking into account all of the above, the results of this study extend our knowledge on another recombinant protein with potential diagnostic utility.

To summarize, this report presents results demonstrating, for the first time, that the full-length AMA1 protein has a better diagnostic potential than the C- or N-terminal fragments of the antigen. The recombinant protein corresponding to amino acid residues 67–569 of the native AMA1 antigen can strongly interact with both anti-*T. gondii* IgG and IgM antibodies, and can be successfully used to develop new diagnostic tools for toxoplasmosis detection. The knowledge gained in this work provides valuable information on the diagnostic utility of the *T. gondii* AMA1 protein, which can be used, inter alia, in the construction of recombinant chimeric antigens, which are considered to be the next generation of diagnostic tools. Nevertheless, before utilizing the AMA1 protein in the clinical diagnosis of *T. gondii* infection, more assays using a larger pool of sera and involving other research laboratories are required. However, it should be highlighted that the present results are very promising.

## 4. Materials and Methods 

### 4.1. Ethics Statement

All procedures used while conducting experiments on mice were approved and carried out according to the guidelines of the Polish Local Ethics Commission for Experiments on Animals No. 9 in Lodz (Agreement 75/ŁB639/2012).

Serum samples tested in the study were obtained during routine toxoplasmosis screening of female volunteers from the area of Pomeranian Voivodeship (Poland). To ensure the anonymity of subjects, only the date of serum collection and the individual’s immune status in regard to anti-*T. gondii* specific antibodies and IgG avidity were disclosed.

### 4.2. Mice

Ten–twelve-week-old female BALB/c and C57BL/6 mice differing in their natural susceptibility to *T. gondii* invasion (BALB/c – more resistant and C57BL/6 – more susceptible) were bred under conventional conditions in the animal facility of the Faculty of Biology and Environmental Protection, University of Lodz, in line with guidelines from the Polish Local Ethics Commission for Experiments on Animals No. 9 in Lodz.

### 4.3. Parasite

The high-virulence *T. gondii* RH strain (ATCC^®^ 50174™) was maintained in vitro in Hs27 cells (ATCC^®^ CRL-1634™), according to the American Type Culture Collection (ATCC) protocol. The RH tachyzoites were used for the preparation of the native *T. gondii* antigen (TLA, 4.4.) and as a source of DNA for cloning experiments. 

The low-virulence DX cyst-forming strain of *T. gondii* used for the induction of experimental murine toxoplasmosis was maintained in vivo in laboratory mice.

### 4.4. Preparation of T. gondii Native Lysate Antigen (TLA)

The *Toxoplasma* lysate antigen, used as a source of the parasite’s native antigens, was prepared as described previously [34], from repeatedly frozen-thawed tachyzoites of the *T. gondii* RH strain.

### 4.5. T. gondii Infection of Mice

BALB/c mice were infected intraperitoneally with five tissue cysts of the *T. gondii* DX strain, which were obtained from the chronically infected C57BL/6 mouse, as described previously [35]. Immune sera (6 per time point) were isolated during acute and late acute toxoplasmosis (2 and 3 weeks post-infection, respectively) and in chronic toxoplasmosis (6 and 12 weeks post-infection). The negative group consisted of six BALB/c mice of a corresponding age and gender, uninfected with *T. gondii*.

### 4.6. IgG and IgM ELISA of Mouse Serum Samples

To assess the reactivity of anti-*T. gondii* IgM and IgG antibodies from mouse sera with the native (TLA) and recombinant antigens, the immunoenzymatic assay was used, as described previously [17]. Briefly, tested antigens, native and recombinant, were used to coat MaxiSorp plates (Nunc) overnight at the concentrations of 10 and 2.5 µg/mL, respectively. Immune and control mouse sera were screened at a 1:100 dilution and goat anti-mouse IgM and IgG immunoglobulins conjugated with horseradish peroxidase (HRP) (Jackson ImunoResearch) were used as secondary antibodies (1:5000). The cut-off values, representing the reactivity of negative controls with each antigen tested, were calculated as the mean absorbance plus two standard deviations. The reactivity of each immune serum with the given antigen was considered positive if the obtained absorbance was higher than the respective cut-off value.

### 4.7. T. gondii RNA Isolation and cDNA Synthesis

The procedures for total RNA extraction and single strand cDNA synthesis were described previously [17].

### 4.8. Construction of the Recombinant Plasmid

The DNA-encoding AMA1 antigen fragments were amplified from the cDNA using a standard PCR amplification protocol with the Phusion High-Fidelity DNA Polymerase (Thermo Fisher Scientific, Inc.). The DNA fragments of the *ama1* (GenBank: XM_002364813.1) gene were obtained by means of PCR using the primers shown in Table 5. The final PCR products were inserted into the *Bgl*II and *Xho*I sites (*ama1* and *ama1C*) or into the *Bgl*II and *Eco*RV sites (*ama1N*) of the pET30 Ek/LIC vector (Novagen) using the In-Fusion® HD Cloning Kit (Takara Bio USA, Inc.). The resulting recombinant plasmids, pET30/AMA1N (containing amino acid residues 67–287 of AMA1), pET30/AMA1C (containing amino acid residues 287–569 of AMA1) and pET30/AMA1 (containing amino acid residues 67–569 of AMA1), were embedded in a frame between the His_6_-tag domains for purification of the recombinant proteins by means of metal affinity chromatography.

### 4.9. Expression and Purification of the Chimeric Antigens and Recombinant Proteins

*E. coli* strain Rosetta (DE3) pLysS transformed with recombinant plasmids pET30/AMA1N, pET30/AMA1C, or pET30/AMA1 was grown in lysogeny broth (LB) medium supplemented with 20 µg/mL of kanamycin and 34 µg/mL of chloramphenicol overnight at 30 °C. Expression was carried out analogously, as described previously [17]. The cells were disrupted by sonication, the insoluble debris was removed by centrifugation, and the protein was purified from the supernatant with the use of a Ni^2+^-iminodiacetic acid-Sepharose column, in accordance with the manufacturer’s instructions (Novagen). The AMA1 and AMA1C proteins were purified in buffers with pH 7.9, while AMA1N was purified at pH 9.5.

The recombinant proteins were analyzed by means of SDS-PAGE on 12% acrylamide gels and stained with Coomassie blue. The concentrations of purified proteins were determined using the Bradford method, with bovine serum albumin as the standard.

### 4.10. Human Serum Samples

The pool of 287 sera was divided into appropriate groups and subgroups based on the commercial test results, as described previously [17]. The serum sample groups used in the IgM and IgG ELISAs are shown in Table 6.

### 4.11. IgG and IgM ELISA–Human Serum Samples

For IgG ELISA tests, MaxiSorp multiwell plates (Nunc) were coated with recombinant proteins AMA1N, AMA1C, and AMA1 or with a TLA at final concentrations of 2.5 μg/mL or 1 μg/mL, respectively. For IgM ELISA assays, MaxiSorp multiwell plates were only coated with AMA1 or with TLA at final concentrations of 10 μg/mL. The concentrations of the recombinant and native antigens were determined in preliminary tests. The subsequent test steps were carried out analogously, as described previously [17].

A positive result was defined as any value higher than the mean OD reading plus two standard deviations (cut-off) obtained with 17 (IgG ELISA) or 20 (IgM ELISA) serum samples from the control group of seronegative samples.

In the IgG ELISA, the calculated cut-off values were 0.745 for the AMA1N, 0.418 for the AMA1C, 0.537 for the AMA1, and 0.409 for the TLA. In the IgM ELISA, the cut-off values were 0.372 for the AMA1 and 0.848 for the TLA.

### 4.12. Statistical Analysis

Statistical analysis was performed using SigmaPlot 14.0 software (Systat Software), as described previously [17].

## Figures and Tables

**Table 1:** The analysis of IgM and IgG antibody levels in the progress of *T. gondii* infection in BALB/c mice.

Antigen	Time after Challenge (Weeks)	IgM		IgG	
		Median	*p*-Value	Median	*p*-Value
**AMA1N**	0	0.165	-	0.118	-
	2	0.411	0.021	0.270	0.002
	3	0.328	0.041	0.594	0.002
	6	0.184	0.485	1.512	0.002
	12	0.223	0.065	1.235	0.002
**AMA1C**	0	0.110	-	0.091	-
	2	0.284	<0.001	0.188	0.002
	3	0.217	<0.001	0.403	0.004
	6	0.201	<0.001	1.404	<0.001
	12	0.181	<0.001	1.167	0.002
**AMA1**	0	0.162	-	0.125	-
	2	1.118	<0.001	1.390	<0.001
	3	0.560	0.002	1.619	0.002
	6	0.264	0.024	1.910	<0.001
	12	0.231	0.065	1.809	<0.001
**TLA**	0	0.318	-	0.159	-
	2	2.784	<0.001	2.392	<0.001
	3	2.018	0.002	2.539	0.002
	6	1.343	0.002	2.732	<0.001
	12	0.862	<0.001	2.846	<0.001

**Table 2:** IgG ELISA of recombinant antigens and TLA can discriminate among samples from suspected acute (I) and chronic (II) phases of *T. gondii* infection patient groups vs. the control (III) group.

Antigen	Calculated Cut-off	ROC Cut-off	AUC	Sensitivity, %	Specificity, %
**AMA1N**	0.7454	0.7453	0.811	36.6	100
**AMA1C**	0.4185	0.4208	0.953	84.6	100
**AMA1**	0.5371	0.5043	0.998	99.4	100
**TLA**	0.4092	0.4130	1.000	100	100

Serum groups used in the IgG ELISA test are described in Section 4.10. Sensitivity and specificity were determined using the cut-off value obtained by ROC analysis for the best discrimination.

**Table 3:** Pearson product-moment correlation coefficient (*r*) for results obtained for recombinant antigens vs. TLA results for different patient groups.

ELISA Test	Group	AMA1N		AMA1C		AMA1	
		*r*	*p* Value	*r*	*p* Value	*r*	*p* Value
**IgM**	I	-	-	-	-	0.449	<0.001
	II	-	-	-	-	0.539	<0.001
	III	-	-	-	-	0.536	<0.001
	*All*	-	-	-	-	0.781	<0.001
**IgG**	I	0.033	0.814	0.529	<0.001	0.382	0.004
	IIA	0.208	0.440	0.075	0.782	0.530	0.035
	IIB	0.514	<0.001	0.154	0.291	0.376	0.008
	IIC	0.339	0.011	0.449	0.001	0.311	0.021
	IIA-C	0.344	<0.001	0.378	<0.001	0.574	<0.001
	III	0.104	0.274	0.195	0.039	0.176	0.063
	*All*	0.587	<0.001	0.523	<0.001	0.793	<0.001

Serum groups used in the IgM and IgG ELISA test are described in Section 4.10.

**Table 4:** The analysis of IgM and IgG antibody levels in the different serum groups.

Antigen	IgM			IgG		
	Group	Median	*p*-Value	Group	Median	*p*-Value
**AMA1N**	-	-	-	I	0.524	<0.001
				IIA	0.755	<0.001
				IIB	0.779	<0.001
				IIC	0.685	<0.001
				III	0.459	-
**AMA1C**	-	-	-	I	0.928	<0.001
				IIA	1.226	<0.001
				IIB	0.774	<0.001
				IIC	0.562	<0.001
				III	0.245	-
**AMA1**	I II III	0.558 0.271 0.216	<0.001 0.014 -	I	1.324	<0.001
				IIA	2.035	<0.001
				IIB	1.919	<0.001
				IIC	1.279	<0.001
				III	0.264	-
**TLA**	I II III	1.392 0.770 0.431	<0.001 <0.001 -	I	0.650	<0.001
				IIA	1.478	<0.001
				IIB	1.301	<0.001
				IIC	0.913	<0.001
				III	0.345	-

Serum groups used in the IgM and IgG ELISA test are described in Section 4.10.

**Table 5:** Oligonucleotide primers used for the amplification of *ama1* gene fragments.

Gene Fragment	Primer Sequence	Corresponding Protein Residues/Plasmid Size
*ama1N*	5’-TGGACAGCCCAGATCGCACGTCGGGGAATCCC-3’ 5’-GAATTCGGATCCGATATCTTTGGGCATTTACTGATGAA CGCATCTG-3’	67–287 (221 aa)/6057 bp
*ama1C*	5’-TGGACAGCCCAGATCCAAATCAAGCTCTTCGCGG GTACAG-3’ 5’-GGTGGTGGTGCTCGAAGTAATCCCCCTCGACCATAACA-3’	287–569 (283 aa)/6192 bp
*ama1*	5’-TGGACAGCCCAGATCGCACGTCGGGGAATCCC-3’ 5’-GGTGGTGGTGCTCGAAGTAATCCCCCTCGACCATAACA-3’	67–569 (503 aa)/6855 bp

**Table 6:** Serum sample groups used in the IgM and IgG ELISAs.

ELISA	Group of Serum Samples	Number of Serum Samples	Total
**IgM**	I (IgG+; IgM+; low avidity–suspected acute phase)	40	156
	II (IgG+; IgM-; high avidity–chronic phase)	40	
	III (IgG-; IgM-–not infected individuals)	76	
**IgG**	I (IgG+; IgM+; low avidity–suspected acute phase)	55	287
	II (IgG+; IgM-; high avidity–chronic phase)	120	
	IIA (IgG > 300 IU/mL)	16	
	IIB (IgG 101–300 IU/mL)	49	
	IIC (IgG ≤ 100 IU/mL)	55	
	III (IgG-; IgM-–not infected individuals)	112	
